# Swiprosin-1 deficiency in macrophages alleviated atherogenesis

**DOI:** 10.1038/s41420-021-00739-y

**Published:** 2021-11-10

**Authors:** Ling-Chang Tong, Zhi-Bin Wang, Jia-Qi Zhang, Yue Wang, Wei-Ye Liu, Hao Yin, Jia-Cheng Li, Ding-Feng Su, Yong-Bing Cao, Li-Chao Zhang, Ling Li

**Affiliations:** 1 Institute of Vascular Disease, Shanghai TCM- Integrated Hospital, Shanghai University of Traditional Chinese Medicine, Shanghai, China; 2 Department of Pharmacology, College of Pharmacy, Naval Medical University, Shanghai, China; 3 Department of Critical Care Medicine, Faculty of Anesthesiology, Naval Medical University, Shanghai, China; 4 Department of Pharmacy, Shanghai Municipal Hospital of Traditional Chinese Medicine, Shanghai, China

**Keywords:** Gene regulation, Cell migration, Atherosclerosis

## Abstract

Macrophages play a vital role in the development of atherosclerosis. Previously, we have found that swiprosin-1 was abundantly expressed in macrophages. Here, we investigated the role of swiprosin-1 expressed in macrophages in atherogenesis. Bone marrow transplantation was performed from swiprosin-1-knockout (*Swp*^*−/−*^) mice and age-matched *ApoE*^*−/−*^ mice. Atherosclerotic lesion, serum lipid, and interleukin-β (IL-β) levels were detected. In vitro, the peritoneal macrophages isolated from *Swp*^*−/−*^ and wild-type mice were stimulated with oxidized low-density lipoprotein (ox-LDL) and the macrophage of foam degree, cellular lipid content, apoptosis, inflammatory factor, migration, and autophagy were determined. Our results showed that swiprosin-1 was mainly expressed in macrophages of atherosclerotic plaques in aorta from *ApoE*^*−/−*^ mice fed with high-cholesterol diet (HCD). The expression of swiprosin-1 in the foaming of RAW264.7 macrophages gradually increased with the increase of the concentration and time stimulated with ox-LDL. Atherosclerotic plaques, accumulation of macrophages, collagen content, serum total cholesterol, LDL, and IL-β levels were decreased in *Swp*^−^^*/*−^ → *ApoE*^*−/−*^ mice compared with *Swp*^*+/+*^ → *ApoE*^*−/−*^ mice fed with HCD for 16 weeks. The macrophage foam cell formation and cellular cholesterol accumulation were reduced, while the lipid uptake and efflux increased in macrophages isolated from *Swp*^−*/−*^ compared to wild-type mice treated with ox-LDL. Swiprosin-1 deficiency in macrophages could inhibit apoptosis, inflammation, migration, and promote autophagy. Taken together, our results demonstrated that swiprosin-1 deficiency in macrophages could alleviate the development and progression of AS. The role of swiprosin-1 may provide a promising new target for ameliorating AS.

## Introduction

Atherosclerosis, characterized by chronic inflammation and abnormal lipid accumulation in the arterial wall, is the major cause of cardiovascular disease [1]. Macrophages are the most abundant immune cells within plaques, originating from circulating monocytes that bind activated endothelial cells and migrate into the intimal layer, as well as from local proliferation of resident macrophages [2]. Macrophages play a pivotal role in the pathogenesis of atherosclerosis by modulating inflammatory status and by scavenging and accumulating excess lipid to become foam cells [3]. Recently, macrophage apoptosis and autophagy have been found to contribute to the formation of the necrotic core and atherosclerotic plaque stability [4]. However, the precise mechanisms that regulate macrophage inflammation, lipid uptake and efflux, apoptosis, autophagy, and migration in the development and progression of atherosclerosis remain largely unresolved.

Swiprosin-1, also known as EF-hand domain-containing 2, is a novel Ca^2+^ sensor protein, which was initially identified in human CD8^+^ T, CD4^+^ T lymphocytes [5] and subsequently in immature, resting, or activated B cells, mast cells, macrophages, natural killer (NK) cells, and platelet and involved in the various cellular functions [6–12]. It has been demonstrated that swiprosin-1 has a role in apoptosis, cell motility, spreading, migration, invasion, metastasis, actin-bundling or cross-linking, immune cell activation, brain cell functions, and so on [6, 13–18]. Its expression is upregulated in many acute and chronic inflammatory diseases such as sepsis [19], passive cutaneous anaphylaxis and atopic dermatitis [9], Alzheimer’s disease [20, 21], dementia [22], schizophrenia [16, 23], diabetic nephropathy [24, 25], and cancer [15, 26, 27], suggesting that swiprosin-1 played a role in inflammation processes of many diseases.

Significantly, our previous study showed that swiprosin-1 was expressed in the peritoneal macrophages and the RAW264.7 cells and upregulated under lipopolysaccharide (LPS) exposure [19, 28]. Moreover, swiprosin-1 was found to directly regulate actin polymerization and participate in LPS-stimulated macrophage migration [28]. Furthermore, our previous study showed that swiprosin-1 deficiency impaired macrophage immune response to LPS-induced or cecal ligation and puncture-induced septic mice [19]. Taken together, our results indicated that swiprosin-1 was expressed in macrophages and involved in acute and chronic inflammation diseases. Macrophages are known to be involved during atherosclerosis development and be the predominant cell type that accumulates cholesterol in the plaque. Atherosclerosis is increasingly recognized as a chronic inflammatory disease. Thus, it is easy to speculate that swiprosin-1 may play a role during the development and progression of atherosclerosis.

Therefore, the aim of the present study was to investigate the role of swiprosin-1 in atherosclerosis. In this study, first, we observed that the expression and distribution of swiprosin-1 in atherosclerotic plaque of mice. Second, the effects of swiprosin-1 deficiency in bone marrow-derived monocytes/macrophages in the development and progression of atherosclerosis were investigated in *ApoE*^*−/−*^ mice fed with a high-cholesterol diet (HCD). Third, the effects of swiprosin-1 on macrophage inflammation, lipid uptake and efflux, apoptosis, autophagy, and migration stimulated with oxidized low-density lipoprotein (ox-LDL) were studied.

## Materials and methods

### Materials

The main materials used in this study were ox-LDL and Dil fluorescently labeled ox-LDL (Dil-ox-LDL) (Xiesheng Biotechnology, Beijing, China); Oil Red O (Santa Cruz Biotechnology Inc., Santa Cruz, CA, USA); Annexin V-FITC apoptosis detection kit and TUNEL apoptosis assay kit (KeyGen Biotech, Jiangsu, China); interleukin-1β (IL-1β) and tumor necrosis factor-α (TNF-α) ELISA kit (Invitrogen, Carlsbad, CA, U.S.A); Trizol reagent (Life Technologies Inc., Gaithersburg, MD, USA); FastStart Universal SYBR-Green Master (Rox) (Roche Diagnostics GmbH, Mannheim, Germany); PrimeScript Reverse Transcription (RT) Master Mix Perfect Real-Time kit (Takara Biotechnology Co., Dalian, China); total cholesterol and LDL assay kit (Jiancheng, Nanjing, China); cholesterol efflux assay kit (Abcam, Cambridge, MA, USA).

The main reagents used in this study were antibodies for swiprosin-1, F4/80, and Bcl-2 (Abcam, Cambridge, England); Bax, caspase-9, light chain 3B (LC3B), and P62 (Cell Signaling Technology, Danvers, MA, USA); caspase-3, β-actin, GAPDH, and Tubulin (Santa Cruz Biotechnology, Inc., Santa Cruz, CA, USA).

### Animals

Male *ApoE*^*−/−*^ mice (4-week-old) were purchased from Beijing University Laboratory Animal Center (Beijing, China). C57BL/6J male mice weighing 18–22 g were obtained from SLRC Laboratory Animal Lid (Shanghai, China). The swiprosin-1 knockout (KO) mice were generated as described previously [29].

All mice were kept under an automated 12-h dark–light cycle at a controlled temperature of 22 ± 2 °C and relative humidity of 50–60% with free access to standard dry diet and tap water ad libitum. All animals received humane care and experimental procedures were performed in accordance with the guidelines of the Naval Medical University for the health and care of experimental animals.

Accelerated atherosclerosis was induced by feeding mice for 4 months with HCD containing (60% raw grain, 16.4% lard, 1.3% cholesterol, 10% sucrose, 0.3% sodium cholate, 8.5% casein protein, 1.9% maltodextrin, 1.6% premix compound). In the end, all animals were sacrificed and blood samples, as well as aortic specimens, were obtained.

### Irradiation and bone marrow transplantation

Eight-week-old male *ApoE*^−^^*/−*^ mice were lethally irradiated with 1000 rads (8 Gy). Bone marrow was collected from femurs of donor *swiprosin-1*^*+/+*^ or *swiprosin-1*^*−/−*^ mice. Irradiated recipient mice were injected with 2 × 10^6^ freshly prepared sterile bone marrow cells via the tail vein. Four weeks after bone marrow transplantation, peripheral blood was collected by retro-orbital venous plexus puncture for polymerase chain reaction (PCR) analysis of bone marrow reconstitution. These chimeric mice were then fed with an HCD diet for 16 weeks to promote atherosclerosis development.

### Histology, immunohistochemistry, and morphometric analyses

Aortas were collected free of connective tissue and fat from the base of ascending aorta to the iliac bifurcation. To quantify the extent of the atherosclerotic lesions in the whole aorta, the aortic arch and thoracic aorta were opened longitudinally, stained with Oil Red O, and pinned on a black wax surface. The percentage of the plaque area stained by Oil Red O compared with the total luminal surface area was quantified blinded by microscopy and computer-aided morphometry. The plaque load was expressed as the percentage of the total surface of the aorta according to previous reports [30].

To quantify the extent of the atherosclerotic lesions in the aortic root, approximately three serial cross sections (5-μm-thick) of the aortic root were prepared. Briefly, atherosclerotic lesions in the aortic sinus region were examined at three locations, each separated by 100 μm, with the most proximal site starting after the appearance of at least two aortic valve leaflets. Every third slide from the serial sections was stained with hematoxylin and eosin (HE), and each consecutive slide was stained with Oil Red O for quantification of the lesion area. CD68 staining was used as a macrophage marker using consecutive slides from serial sections. Collagen content was assessed by Masson’s trichrome of consecutive slides from serial sections. All images were acquired using the OLYMPUS IX71 microscope. Quantitative analysis of staining was performed blinded by two observers with Image-Pro Plus software.

### Layer separation of aorta

Manual layer separation of the aorta was performed as described previously [31, 32]. The aorta was isolated and washed at 4 °C in a physiological saline solution (0.9% w/v of NaCl). All samples were subjected to a standardized preparation procedure, consisting of the mechanical removal of the periaortic adipose and connective tissue and of the protruding portion of the aortic branches under a dissection microscope with surgical scissors and forceps. The artery was opened longitudinally and endothelium faced upwards and then was gently flushed with ice-cold phosphate-buffered saline (PBS) to remove the residual blood. Then, the vessel was placed on a glass slide. The intimal surface was rubbed with a cotton swab. This was performed longitudinally across the luminal surface of the artery five times in each direction. The cotton swab was rinsed three times in cold PBS and the liquid containing the endothelial cells was centrifuged. The cell pellet was collected and then lysed with RIPA buffer for western blotting. The medial and adventitial layers were then separated mechanically. The mechanical removal of the adventitia was attempted; outer layers of the vessel wall were gradually stripped bluntly using surgical tweezers with hooks or one blade of scissors until it was not possible to remove further tissue layers without destruction of the artery. The remaining part was the medial layer. Afterwards, the stripped segments and medial layer were processed, respectively, for western blotting.

### Serum lipid and cytokine assay

No-fasting serum lipids, including total cholesterol and LDL cholesterol, were determined using total cholesterol and LDL assay kit according to the manufacturer’s instructions.

Serum IL-1β and TNF-α were measured using mouse IL-1β and TNF-α ELISA kits according to the manufacturer’s instructions, respectively. The optical density was measured at 450 nm. Finally, the cytokine concentrations were calculated using the standard curve obtained in each experiment.

### Preparation of the peritoneal macrophages

The peritoneal macrophages were obtained from the mice after intraperitoneal injection of 3 mL of 3% thioglycolate as described previously [19]. Briefly, the mice were sacrificed and the macrophages were isolated by lavage with 5 mL of RPMI (Gibco), washed twice with PBS after 3 h of adherence, cultured in RPMI at 37 °C and 5% CO_2_, and finally stimulated with ox-LDL. The isolated cells were used for foam cell formation analysis, cholesterol measurement, cytokine analysis, Western blotting, and flow cytometry.

### Cell culture

The RAW264.7 macrophage cell line was purchased from the National Infrastructure of Cell Line Resource (Beijing, China). RAW264.7 cells were cultured in RPMI-1640 medium supplemented with 10% fetal bovine serum (FBS) and 1% penicillin–streptomycin in a humidified incubator with 5% CO_2_ at 37 °C. The cells were treated with ox-LDL at different concentrations and times.

### Immunofluorescence

Immunofluorescence was performed as described previously [33]. Macrophages in eight-well culture slides or aortic sections were fixed with 4% paraformaldehyde in PBS for 10 min. Blocking was performed with immunofluorescence buffer (PBS, 2% bovine serum albumin (BSA), 10% FBS) for 1 h, followed by a 10-min incubation with a second immunofluorescence buffer (PBS, 0.4% Triton X-100). Cells/tissues were incubated with primary antibody against F4/80 (Abcam, 1:200) and swiprosin-1 (Santa Cruz Biotechnology, 1:50) for 3 h at room temperature in a humidified chamber. After washing, cells were incubated with Cy3-labeled goat anti-mouse IgG (H + L) or Alexa Fluor 488-labeled goat anti-rabbit IgG (Beyotime, 1:200) for 1 h. 4′,6-Diamidino-2-phenylindole (DAPI) (Beyotime) was used for nuclear staining. Images were acquired using a fluorescence microscope (Olympus IX71, Olympus, Tokyo, Japan).

### Flow cytometry

To confirm the purity of macrophages, the cells isolated from the peritoneal fluid of the mice were incubated with anti-mouse F4/80-PE (eBioscience, USA) fluorescent antibodies for 30 min and were washed twice with staining buffer before measurement with a BD FACS Calibur flow cytometer (BD Biosciences, East Rutherford, NJ, USA). In brief, cells were captured via high forward scatter and high side scatter. Favorite cells were gated as shown in Region 1 (R1), of which PE-positive cells were selected. Results were expressed in the percentage of positive cells. The experiments were performed in triplicate.

To measure apoptosis, the cells treated with ox-LDL were washed with cold PBS and resuspended in 1 mL of 1× binding buffer, and then 100 μL of cells (1 × 10^5^) was placed into individually labeled tubes. Annexin V-FITC was added to the cellular suspension, as per the manufacturer’s instructions, and a fluorescent sample of 10,000 cells was immediately analyzed with a BD FACS Calibur flow cytometer.

### Cellular cholesterol measurement and foam cell formation analysis

Peritoneal macrophages from WT and *swiprosin-1*^−*/−*^ mice were plated on 12-well plates and incubated with or without ox-LDL (40 μg/mL). After 24 h, intracellular cholesterol content was measured using the Amplex Red Cholesterol Assay kit (Molecular Probes; Invitrogen), according to the manufacturer’s instructions.

For the foam cell formation assay, mouse peritoneal macrophages were grown on coverslips and treated with ox-LDL (40 μg/mL) for 24 h. Cells were fixed with 4% paraformaldehyde for 20 min. After washing three times with PBS, cells were blocked for 30 min (PBS + BSA 1%) and stained with Oil Red O at room temperature. Six random fields per condition were captured with Olympus IX71 microscope and quantification was performed with Image-Pro Plus software.

### TUNEL staining

To detect the typical features of apoptosis, nuclear DNA was stained using terminal deoxynucleotidyl transferase dUTP nick-end labeling (TUNEL). After treatment, peritoneal macrophages were treated with proteinase K (20 μg/mL, 37 °C, 15 min) and incubated with the TUNEL reaction mixture in a moist chamber (dark, 37 °C, 1 h). The cells were counterstained with DAPI to detect the nuclei. TUNEL-positive cells were observed with a fluorescence microscope (Olympus IX71).

### Analysis of Dil-ox-LDL uptake

Dil-ox-LDL was used for the uptake study. Peritoneal macrophages from WT and *swiprosin-1*^*−/−*^ mice were co-incubated with Dil-ox-LDL (50 μg/mL) for 4 h at 37 °C. Fluorescence intensity was detected using Olympus IX71 fluorescence microscope and calculated using the Image-Pro Plus software. Three independent experiments were performed.

### Cholesterol efflux assay

The cholesterol efflux in peritoneal macrophages was examined with the cholesterol efflux assay kit (cell-base) (Abcam, ab196985) according to the manufacturer’s instructions. Briefly, mouse primary peritoneal macrophages (1 × 10^5^ cells/well) were incubated in a 96-well plate. The labeling reagent and equilibration buffer mix (100 μL) was added to each well and cells were incubated for 16 h at 37 °C before ox-LDL was added to each well as a cholesterol acceptor. The supernatant was transferred to a new 96-well plate and fluorescence was measured using a multiwell plate reader (A, Ex/Em = 482/515 nm). The cells were lysed by cell lysis buffer (100 μL/well) and then the plate was shaken for 30 min at room temperature. The cell lysate was transferred to a new 96-well plate and fluorescence was measured (B, Ex/Em = 482/515 nm). The cholesterol efflux was calculated by dividing the A by (A + B) in each group (C = 100% × A/(A + B)). All samples were assayed with three replicates.

### RNA extraction and real-time PCR

Total RNA was extracted from the primary peritoneal macrophages using Trizol reagent and reverse transcribed into cDNA with a PrimeScript RT Master Mix Perfect Real-Time kit. Quantitative real-time PCR was performed using FastStart Universal SYBR-Green Master (Rox) and carried out on an ABI 7500 real-time PCR system (Applied Biosystems, Foster, CA, USA) using the following primers. β-actin forward primer, 5′-GTCCCTCACCCTCCCAAAAG-3′; reverse primer, 5′-GCTGCCTCAACACCTCAACCC-3′. CD36 forward primer, 5′-GATGACGTGGCAAAGAACAG-3′; reverse primer, 5′-TCCTCGGGGTCCTGAGTTAT-3′. Scavenger receptors type A (SR-A) forward primer, 5′-TGGTCCACCTGGTGCTCC-3′; reverse primer, 5′-ACCTCCAGGGAAGCAATTT-3′. β-actin was used as the reference gene. Relative quantitation of target genes was calculated by the 2^−ΔΔCt^ method as recommended by the manufacturer. Corresponding peritoneal macrophages isolated from WT mice were used as control samples for messenger RNA (mRNA) expression of target genes. Each experiment was repeated three times in three samples.

### Protein extraction and immunoblotting analysis

Immunoblotting was performed as described previously [29]. Briefly, the aortic tissue was sliced into sections and washed with PBS. Tissues or cell proteins were lysed in RIPA buffer containing protease and phosphatase inhibitor cocktail. The mixture was centrifuged at 14,000 × *g* and 4 °C for 15 min, and the protein content in the supernatant was determined by the Bradford method. An equal amount of protein was separated by 10% (w/v) sodium dodecyl sulfate-polyacrylamide gel electrophoresis and transferred to nitrocellulose membranes. The membranes were blocked for 3 h at room temperature with blocking reagent and the primary antibodies including anti-swiprosin-1 (1:1000), ABCA1 (1:1000), ABCG1 (1:1000), Bax, Bcl-2, procaspase-3, caspase-9, GAPDH, and Tubulin antibody (1:5000) were incubated overnight at 4 °C. After washing with PBST, the membranes were incubated with corresponding secondary antibodies (1:10,000 dilution) for 50 min at room temperature. Specific bands were scanned and analyzed by Odyssey Infrared Imaging System (LI-COR, Lincoln, Nebraska USA). Tubulin was used as the protein loading control. All immunoblotting experiments were repeated at least three times.

### Transwell migration assay

As previously described before [28], cells were stimulated with or without ox-LDL for 24 h. Cells of each group were added to the upper wells (24-multiwell Corning Costar Transwell) at a density of 1 × 10^5^ to each well. Migrating cells could pass through the polycarbonate filter (8 μm). Cells were fixed in 4% paraformaldehyde for 10 min. The nonmigratory cells were removed from the upper membrane surface carefully with cotton sticks. Then, the cells were counterstained with DAPI to detect the nuclei and imaged by a fluorescence microscope (Olympus IX71). The excitation and emission wavelengths were 358 and 461 nm, respectively. The numbers of migratory cells were counted at high magnification (×400) in ten fields chosen randomly for each membrane.

### Statistical analysis

All the data were expressed as mean ± SE. Unpaired Student’s *t* test was used for comparison of data from two groups. Analysis of variance was used for the comparison of data involving more than two groups. *P* < 0.05 was considered statistically significant. Statistical analysis was performed using the SPSS 22.0 software (SPSS Inc., Chicago, IL, USA).

## Results

### Swiprosin-1 expressed in atherosclerosis plaque

**Fig. 1:**
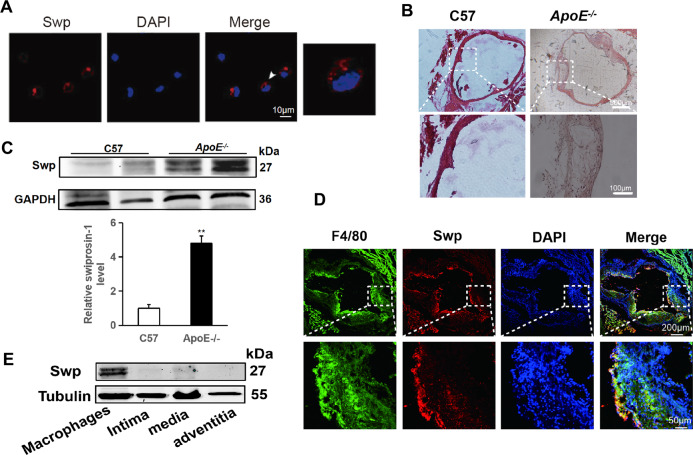
Swiprosin-1 expressed in macrophages of mouse atherosclerosis plague. **A** Representative image of immunofluorescent staining for swiprosin-1 (red) in RAW264.7 macrophages. The localization of the nucleus was detected by DAPI staining (blue). Scale bars are 10 μm (left) and 2 μm (right). **B** Representative images of aortic root sections stained with hematoxylin and eosin. *n* = 3/group. Scale bars are 500 μm (upper) and 100 μm (down). **C** Representative Western blot for swiprosin-1 expression in aorta tissue from C57BL/6 and *ApoE*^*−/−*^ mice fed a high-cholesterol diet for 4 months (upper) and densitometric analysis (down). The experiment was repeated at least three times. GAPDH served as the loading control. Data are presented as the mean ± SE; **P < 0.01. **D** Representative images of immunofluorescent double staining for swiprosin-1 (red) and F4/80 (macrophage marker, green) in atherosclerotic plaque. The localization of the nucleus was detected by DAPI staining (blue). *n* = 6/group. Scale bars are 200 μm (upper) and 50 μm (down). **E** Representative Western blot for swiprosin-1 expression in each layer of the aortic wall. The result was repeated in at least three independent experiments. Tubulin served as the loading control. *ApoE*^*−/−*^ apolipoprotein E-deficient, Swp swiprosin-1, DAPI 4′,6-diamidino-2-phenylindole.

Our previous study has shown that swiprosin-1 was abundantly expressed in macrophages [19, 28]. Immunofluorescence results showed that swiprosin-1 was expressed in the cytoplasm and cell membrane of the macrophage (Fig. 1A). To investigate whether swiprosin-1 was involved in atherosclerosis, the C57 mice and ApoE^−/−^ mice were fed with a western high-fat diet for 4 months. HE staining showed that there were atherosclerotic plaques on the aortic wall of the mice in ApoE^−/−^ mice, indicating that the atherosclerosis model was successful (Fig. 1B). Western blot showed that swiprosin-1 expression in ApoE^−^^/−^ mice was higher than that in C57 mice, indicating that swiprosin-1 expression increased with the development of atherosclerosis (Fig. 1C). To explore the distribution of swiprosin-1 in atherosclerotic plaques, double-labeling immunofluorescence was used with macrophage-specific markers F4/80 and swiprosin-1. The fluorescence in the plaque was relatively high, indicating that swiprosin-1 expression was mainly in the plaque and colocalized with the macrophage in the plaque (Fig. 1D). It was known that the aorta mainly consists of three layers: the tunica intima, media, and adventitia. To further confirm the distribution of swiprosin-1 in macrophage of plaque, the aorta was separated into layers and the intima, media, and adventitia were taken for western blot. Swiprosin-1 was expressed in none of the three layers in the aorta (Fig. 1E). The results further demonstrated that swiprosin-1 was mainly expressed in macrophages of atherosclerotic plaques in the aorta.

### Swiprosin-1 expression increased in macrophages treated with ox-LDL

**Fig. 2:**
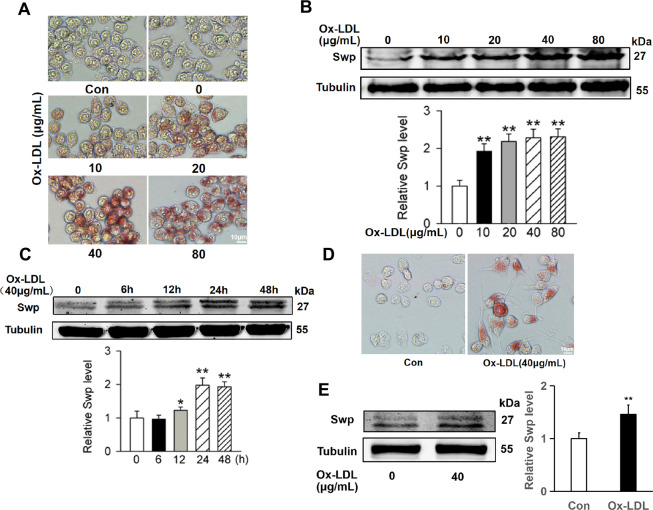
Swiprosin-1 expression increased in macrophage induced by ox-LDL. **A** Representative images of RAW264.7 cells stained with Oil Red O after induced by ox-LDL for 24 h. *n* = 3/group. Scale bars are 10 μm. **B** Representative Western blot for swiprosin-1 expression in RAW264.7 cells induced by different doses of ox-LDL for 24 h (upper) and densitometric analysis of swiprosin-1 expression (down). Tubulin served as the loading control. Data are presented as the mean ± SE; **P < 0.01, *n* = 3/group. **C** Representative Western blot for swiprosin-1 expression in RAW264.7 cells induced by ox-LDL at different time intervals (upper) and densitometric analysis (down). Tubulin served as the loading control. Data are presented as the mean ± SE; **P < 0.01, *n* = 3 per group. **D** Representative images of primary peritoneal macrophages from C57BL/6 mouse stained with Oil Red O after induced by ox-LDL (40 μg/mL) for 24 h. Scale bars are 10 μm. **E** Representative Western blot for swiprosin-1 expression in primary peritoneal macrophages from C57BL/6 mouse induced by ox-LDL for 24 h (left) and densitometric analysis (right). Tubulin served as the loading control. Data are presented as the mean ± SE; **P < 0.01, *n* = 3 per group. Con control, ox-LDL oxidized low-density lipoprotein, Swp swiprosin-1.

It was demonstrated that macrophage-derived foam cells played an important role in the development of atherosclerosis. To investigate whether swiprosin-1 plays a role in the foaming process of macrophage, RAW264.7 cells were stimulated with different concentrations of ox-LDL for 24 h. Oil Red O staining revealed that the lipids accumulation in macrophages increased after ox-LDL stimulation. The lipid content in RAW264.7 cells increased with the increase of ox-LDL concentration, indicating that the foam cell model was successful (Fig. 2A). The expression of swiprosin-1 gradually increased after the foaming of the macrophage with the increase of the concentration of ox-LDL. The expression of swiprosin-1 reached a peak after RAW264.7 cells treated with ox-LDL at 40 μg/mL (Fig. 2B). As shown in Fig. 2C, the expression of swiprosin-1 in RAW264.7 cells gradually increased with time stimulated by ox-LDL. The expression of swiprosin-1 reached a peak after being treated with ox-LDL for 24 h. Furthermore, the primary peritoneal macrophages from the C57 mouse were extracted and verified by flow cytometry staining with F4/80 (Supplement 1). The primary peritoneal macrophages were cultured for 24 h and then treated with 40 μg/mL Ox-LDL for 24 h. Oil Red O staining proved that the foamed cell modeling was successful (Fig. 2D). Western blot results showed that the expression of swiprosin-1 in the primary peritoneal macrophages increased with the foaming of cells (Fig. 2E). These results demonstrated that swiprosin-1 expression increased with the foaming of macrophages induced by ox-LDL.

### Swiprosin-1 deficiency alleviated the development of atherosclerosis

**Fig. 3:**
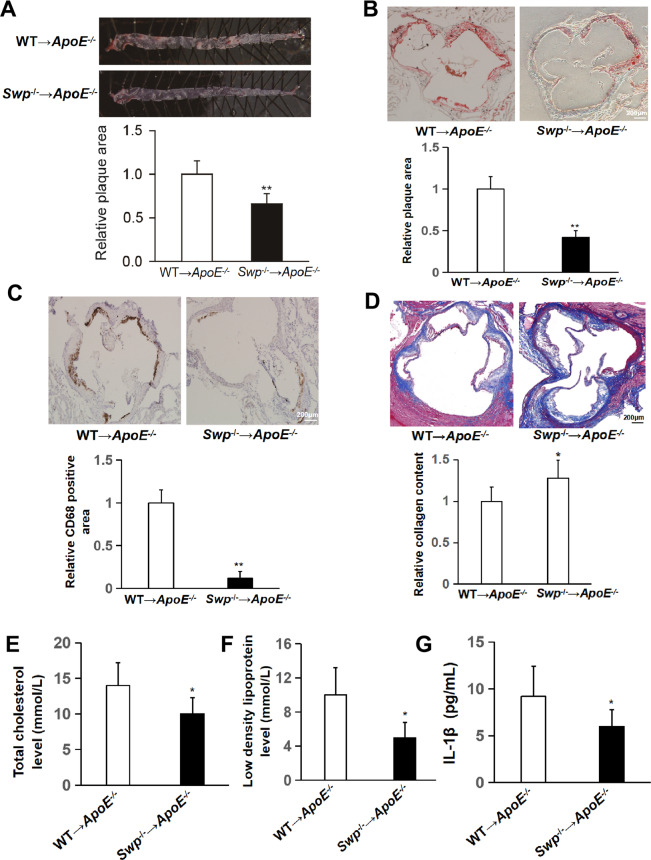
Swiprosin-1 deficiency alleviated atherosclerosis. Atherosclerotic lesion formation was assessed in *ApoE*^−*/−*^ mice transplanted with swiprosin-1^−*/−*^ (*Swp*^−*/−*^) and wild-type mice (WT) bone marrow and fed a high-cholesterol diet for 16 weeks. **A** Representative images of en face aortas from *Swp*^−*/−*^ → *ApoE*^−*/−*^ and WT → *ApoE*^−*/−*^ mice stained with Oil Red O (upper) and quantitative comparison of atherosclerotic lesion area (down). The plaque-covered area was determined and expressed as a percent of the total area. Data are presented as the mean ± SE; **P < 0.01, *n* = 6 per group. **B** Representative images of aortic root sections stained with Oil Red O (upper) and quantitative comparison of atherosclerotic lesion area (down). Data are presented as the mean ± SE; **P < 0.01, *n* = 3 per group. Scale bars are 200 μm. **C** Representative images of macrophages in aortic plaque determined by immunohistochemistry using anti-CD68 antibody (upper) and quantitative comparison of CD68-positive area (down). Data are presented as the mean ± SE; **P < 0.01, *n* = 3 per group. Scale bars are 200 μm. **D** Representative images of collagen content assessed by Masson’s trichrome stain (upper) and quantitative comparison of collagen content (down). Data are presented as the mean ± SE; *P < 0.05, *n* = 3/group. Scale bars are 200 μm. Serum total cholesterol (**E**), low-density lipoprotein (**F**), and IL-1β (**G**) were determined by ELISA kit. Data are presented as the mean ± SE; *P < 0.05, *n* = 6–7/group.

To investigate whether swiprosin-1 expression in macrophages contributed to the progression of atherosclerosis [34], bone marrow transplantation from *swiprosin-1*^*−/−*^ mice as a donor to *ApoE*^*−/−*^ as a recipient and *swiprosin-1*^*+/+*^ mice as a donor to *ApoE*^*−/−*^ as a recipient were performed and then fed with HCD for 4 months. As shown in Fig. 3A, the en face Oil Red O-positive lesion area of the aorta in *Swp*^*−/−*^ → *ApoE*^*−/−*^ group was significantly lower than that in the control group. HE staining of cross sections of aorta root showed that atherosclerotic plaques were typical in *Swp*^*+/+*^ → *ApoE*^*−/−*^ group (Supplement 2). As shown in Fig. 3B, plaque size at the aortic root in *Swp*^*−/−*^ → *ApoE*^*−/−*^ group decreased compared to *Swp*^*+/+*^ → *ApoE*^*−/−*^ group. Collectively, these results showed that swiprosin-1 deficiency alleviated the development of atherosclerosis in mice.

The severity of atherosclerosis is not only related to the size of the plaque but also the stability of the plaque. The stability of the atherosclerotic plaque is closely related to the cellular components in the plaque [35], where the more macrophages, the worse the stability of the plaque [36]. To investigate whether swiprosin-1 affects macrophage accumulation, which is related to plaque stability, CD68 (macrophage)-positive area in atherosclerotic plaques in *Swp*^*−/−*^ → *ApoE*^−^^*/−*^ group was found to be significantly reduced compared with *Swp*^*+/+*^ → *ApoE*^*−/−*^ group (Fig. 3C). Similarly, collagen content (Masson’s trichrome stain) significantly increased in *Swp*^*−/−*^ → *ApoE*^*−/−*^ group (Fig. 3D). These observations are consistent with reduced lesion formation in *Swp*^*−/−*^ → *ApoE*^−^^*/−*^ mice with a phenotypic shift toward decreased features of plaque vulnerability.

Dyslipidemia is an important risk factor that predisposes an individual to develop atherosclerosis [37]. To investigate the effect of swiprosin-1 on serum lipid profile, the levels of serum total cholesterol and LDL in mice were determined. It was shown that the levels of total cholesterol and LDL decreased in *Swp*^*−/−*^ → *ApoE*^*−/−*^ group compared with *Swp*^*+/+*^ → *ApoE*^*−/−*^, respectively (Fig. 3E, F). These findings demonstrated that the absence of swiprosin-1 lowered blood lipid in *Swp*^−^^*/−*^ → *ApoE*^*−/−*^ mice.

Chronic inflammation is part of the pathological process during atherosclerosis [38]. To investigate the effect of swiprosin-1 on inflammation, the level of IL-1β was determined. It was shown that the level of IL-1β in serum was lower in *Swp*^*−/−*^ → *ApoE*^*−/−*^ group compared with *Swp*^*+/+*^ → *ApoE*^*−/−*^ (Fig. 3G).

### Swiprosin-1 deficiency reduced macrophage foam cell formation

**Fig. 4:**
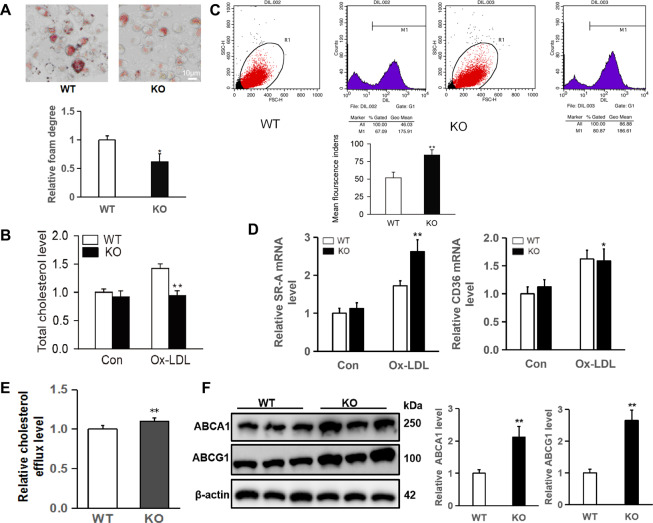
Swiprosin-1 deficiency attenuated foam cell formation and cellular total cholesterol level and enhanced the lipid uptake and efflux. **A** Representative images of primary peritoneal macrophages from swiprosin-1^−*/−*^ (*Swp*^−*/−*^) and wild-type (WT) mice stained with Oil Red O after stimulated with ox-LDL (40 μg/mL) for 24 h (upper) and quantitative analysis of foamy macrophages (down). Data are presented as the mean ± SE; *P < 0.05, *n* = 3 per group. Scale bars are 10 μm. B Total cholesterol levels in primary peritoneal macrophages of *Swp*^−*/−*^ and WT mice after stimulated with ox-LDL for 24 h were compared. Data are presented as the mean ± SE; **P < 0.01, *n* = 3 per group. **C** Dil-ox-LDL uptake by primary peritoneal macrophages from *Swp*^−*/−*^ and WT mice was detected by flow cytometry. Data are presented as the mean ± SE; **P < 0.01, *n* = 3 per group. **D** The SR-A and CD36 mRNA expression in primary peritoneal macrophages was quantitated by real-time PCR. Data are presented as the mean ± SE; *P < 0.05, **P < 0.01; *n* = 3 per group. **E** The relative levels of cholesterol efflux were compared. Data are presented as the mean ± SE; *P < 0.05, *n* = 6/group. **F** Representative Western blot for ABCA1 and ABCG1 expression in primary peritoneal macrophages (left) and densitometric analysis (right). β-Actin served as the loading control. Data are presented as the mean ± SE; **P < 0.01; *n* = 3 per group.

To investigate the effect of swiprosin-1 on the foaming of macrophages, the primary peritoneal macrophages from *Swp*^*−/−*^ mice and wild-type mice were extracted and cultured, and then stimulated with ox-LDL for 24 h. Oil Red O staining revealed a significant reduction of neutral lipid accumulation in swiprosin-1-deficient macrophages compared with WT macrophages. The relative foam degree was decreased in macrophages from swiprosin-1 KO mice compared with that from WT mice (Fig. 4A). Similarly, the cellular total cholesterol content in macrophages was found to be decreased after swiprosin-1 KO (Fig. 4B).

We next investigated whether the reduced lipid accumulation observed in the absence of swiprosin-1 was due to reduced uptake of modified lipoproteins or increased cellular cholesterol efflux.

In order to study the effect of swiprosin-1 on lipid uptake, macrophages were stimulated with Dil-ox-LDL and then detected by flow cytometry. It was noteworthy that the uptake of LDL by macrophages was enhanced after swiprosin-1 KO (Fig. 4C). The mRNA expression of SR-A was enhanced in swiprosin-1 KO macrophages compared to the control macrophages. The expression of CD36 mRNA was no different between swiprosin-1 KO macrophages and control macrophages induced by ox-LDL. Thus, we wondered whether the cellular cholesterol efflux might be increased in swiprosin-1 KO macrophage.

As shown in Fig. 4E, the cholesterol efflux in macrophages increased in swiprosin-1 KO group than in the WT group. Moreover, we further analyzed the protein expression of ATP-binding cassette transporters A1 and G1 (ABCA1 and ABCG1) that control the cellular cholesterol efflux. As expected, ABCA1 and ABCG1 expression was upregulated in swiprosin-1-KO macrophages compared to control macrophages (band densitometry analysis in the down panel) (Fig. 4F).

Collectively, these results demonstrated that swiprosin-1 deficiency in macrophages could not only increase the uptake of lipids but also enhance cholesterol efflux. Our findings suggested that the decrease of lipid accumulation in swiprosin-1 deficiency macrophages might mainly be through enhancing the cholesterol efflux.

### Swiprosin-1 deficiency alleviated macrophage apoptosis

**Fig. 5:**
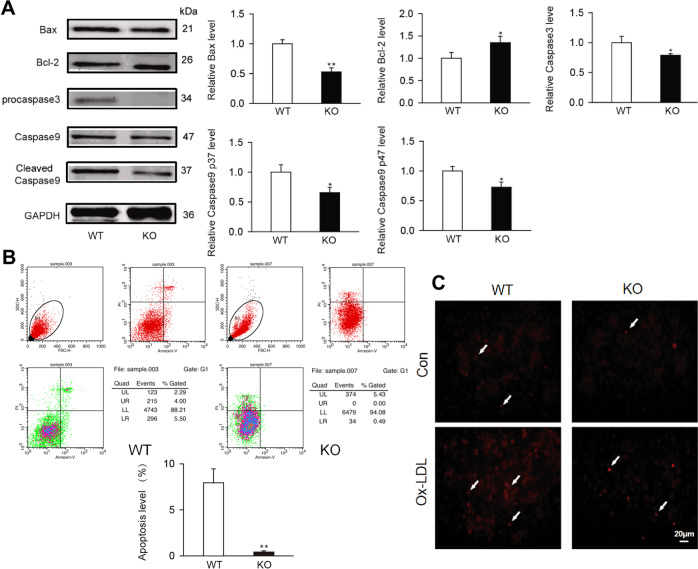
Swiprosin-1 deficiency inhibited macrophages apoptosis. **A** Representative Western blot for ox-LDL induced apoptosis-related protein expression in primary peritoneal macrophages from *Swp*^−*/−*^ and WT mice (left) and densitometric analysis (right). GAPDH served as the loading control. Data are presented as the mean ± SE; *P < 0.05, **P < 0.01; *n* = 3 per group. **B** Apoptosis in primary peritoneal macrophages induced by ox-LDL was detected by Annexin V-FITC/PI apoptosis detection kit using flow cytometry. Data are presented as the mean ± SE; *P < 0.05, *n* = 3 per group. **C** Representative images of apoptosis in primary peritoneal macrophages detected by TUNEL kit (upper) and quantitative analysis (down). Data are presented as the mean ± SE; *P < 0.05, *n* = 3/group.

It was demonstrated that macrophage apoptosis is a crucial determinant of atherosclerosis lesion progression and plaque stability [39]. To study whether swiprosin-1 plays a role in macrophage apoptosis, the expression levels of genes involved in apoptosis were determined. As shown in Fig. 5A, proapoptosis-related proteins such as Bax, caspase-3, and caspase-9 are all down-regulated after swiprosin-1 was knocked out, while the anti-apoptotic protein Bcl-2 was upregulated, suggesting that swiprosin-1 KO in macrophage could inhibit apoptosis. Further, the Annexin V-FITC cell apoptosis detection kit was used to detect the effect of swiprosin-1 in promoting cell apoptosis. The same results were verified by flow cytometry (Fig. 5B).

As shown in Fig. 5C, the apoptotic cells increased after the macrophages were stimulated with ox-LDL using the TUNEL method. The apoptotic cells significantly reduced after swiprosin-1 KO. The results confirmed that swiprosin-1 played a role in promoting apoptosis of macrophages and swiprosin-1 deficiency could inhibit the apoptosis of macrophages.

### Swiprosin-1 deficiency inhibited macrophage migration and inflammation, enhanced macrophage autophagy

**Fig. 6:**
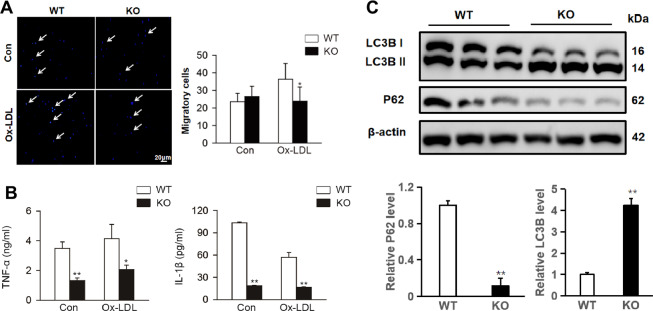
Swiprosin-1 deficiency inhibited cytokine secretion and macrophage migration and promoted macrophage autophagy. **A** The levels of TNF-α and IL-1β were detected by ELISA kit. Data are presented as the mean ± SE; *P < 0.05, **P < 0.01, *n* = 3/group. **B** Primary peritoneal macrophages migration was detected by Transwell migration assay. Data are presented as the mean ± SE; *P < 0.05; *n* = 6/group. **C** Representative Western blot for autophagy-related protein (P62/SQSTM1 and LC3B) in primary peritoneal macrophages of *Swp*^−*/−*^ and WT mice after stimulated with ox-LDL for 24 h (upper) and densitometric analysis (down). β-Actin served as the loading control. Data are presented as the mean ± SE; **P < 0.01. The result was repeated in at least three independent experiments.

The migration capacity of macrophages was assessed using Transwell assay in vitro. There was no significant difference in the number of migratory cells between peritoneal macrophages isolated from WT and KO mice. However, the number of migratory cells decreased in swiprosin-1 KO-derived macrophages compared with the WT-derived macrophages treated with ox-LDL, indicating that swiprosin-1 deficiency inhibited macrophage migration (Fig. 6A).

As shown in Fig. 6B, the level of TNF-α and IL-1β in cell supernatant was lower in *Swp*^*−/−*^ group compared with *Swp*^*+/+*^ group after being stimulated with ox-LDL for 24 h.

Macrophage autophagy has been shown to play a protective role in atherosclerosis. As an indicator of autophagic activity, microtubule-associated proteins 1 LC3B II/I ratio increased in swiprosin-1 KO macrophages compared with WT macrophages. Moreover, P62/SQSTM1 level correlates with autophagic activation, and an entire autophagic flux decreased in macrophages after swiprosin-1 KO (Fig. 6C). The results indicated that swiprosin-1 deficiency might enhance macrophage autophagy.

## Discussion

In the current study, we found that swiprosin-1 was highly expressed in macrophages in atherosclerotic plaque and upregulated in both in vivo model aortic tissues of atherosclerosis and in vitro RAW264.7 cells or cultured mouse peritoneal macrophages induced by ox-LDL. We demonstrated that swiprosin-1 deficiency in macrophages could inhibit the progression of atherosclerosis. We also found that swiprosin-1 deficiency could inhibit apoptosis, inflammation, and migration and promote the uptake of lipid, cholesterol efflux, and autophagy in macrophages stimulated with ox-LDL.

Swiprosin-1 has originally been found to be involved in lymphocytes cytotoxicity and expressed in diverse cell types [40]. Our previous studies demonstrated that swiprosin-1 played an important role in the macrophage immune response to sepsis and was involved in LPS-stimulated macrophage migration [19, 28]. Here, we found that swiprosin-1 was expressed in mouse atherosclerotic plaques and swiprosin-1 deficiency in bone marrow-derived macrophages alleviated atherosclerotic plaque formation. The underlying mechanisms might be that swiprosin-1 deficiency decreased foam cell formation and cellular cholesterol accumulation mainly through enhancing the cholesterol efflux. Moreover, our results indicated that swiprosin-1 deficiency in macrophages could suppress apoptosis (Bax, Bcl-2, procaspase-3, and caspase-9), inflammation (TNF-α and IL-β), and migration and enhanced autophagy (LC3B and P62). Therefore, we demonstrated that swiprosin-1 deficiency might play a protective role during foam cell formation and atherosclerosis. Indeed, swiprosin-1 was also found to be in many other immune cells including CD8^+^ T, CD4^+^ T lymphocytes [5], NK cells [41], and mast cells [8, 9], which have emerged as critical drivers and modifiers of the pathogenesis of atherosclerosis [42, 43]. The mouse model of bone marrow transplantation could not specifically distinguish the role of swiprosin-1 in macrophages in the development of atherosclerosis. Thus, the role of specific deletion swiprosin-1 in macrophage in the development of atherosclerosis needs further study.

Interestingly, our results showed that swiprosin-1 deficiency in macrophages increased the uptake of lipids as well as the cholesterol efflux. We assumed that the decrease of cholesterol accumulation in swiprosin-1 deficiency macrophages treated with ox-LDL may mainly be due to enhancing the cholesterol efflux. Our results showed that the expression of transporters ABCA1 and ABCG1 in macrophages increased after swiprosin-1 KO. ABCA1 and ABCG1 expression associates well with the lesion severity and progression [44]. It was shown that the inactivation of macrophage ABCA1 promotes increased atherosclerosis in ApoE-deficient mice [45]. However, a recent study showed that higher levels of swiprosin-1 and ABCC1 were found in cisplatin-resistant CL1-0 cells compared with the parental cells, indicating that swiprosin-1 promoted cisplatin resistance through activating ABCC1 for drug efflux in non-small cell lung cancer [27]. The reasons why our results are inconsistent with this study may be due to the different cell types used in the experiment. The effect of swiprosin-1 on the transporters may be different in macrophages and non-small cell lung cancer cells. However, the mechanism of swiprosin-1 which regulates intracellular cholesterol concentration via cholesterol transporters in macrophages needs to be further studied.

It was reported that ectopic expression of swiprosin-1 in the murine B cell line WEHI231 enhanced spontaneous and BCR-induced apoptosis with loss of the mitochondrial membrane potential [6]. Swiprosin-1 was found to bind to caspase-9 in human H460 non-small lung cancer cells, indicating that swiprosin-1 might be involved in caspase-9 dependent apoptosis [7]. Our previous study also showed that over-expression of swiprosin-1 in human glomerular endothelial cells increased apoptosis and promoted the formation of apoptosomes [24]. Moreover, our previous study showed that swiprosin-1 deficiency attenuated mitochondria-dependent podocytes apoptosis in early diabetic nephropathy [25]. Consistent with these findings, we demonstrated that swiprosin-1 deficiency attenuated apoptosis in peritoneal macrophages treated with ox-LDL in vitro. It was known that macrophage apoptosis occurred during all stages of atherosclerosis and influences early lesion formation, plaque progression, and plaque stability. In our study, mice were fed with HCDs for 4 months, which should be the progression of atherosclerosis. Macrophage apoptosis in atherosclerotic lesions is a major contributor of atherosclerotic plaque instability, rupture, and thrombus formation. Thus, our findings provided evidence that swiprosin-1 in macrophages could aggravate plaque instability in the progression of atherosclerosis. In our study, to confirm that swiprosin-1 in macrophages regulated atherosclerosis, bone marrow transplantation was performed. It was demonstrated that swiprosin-1 deficiency in bone marrow-derived cells including monocytes/macrophages alleviated atherosclerosis. However, it was demonstrated that local macrophage proliferation is the dominant cause of macrophage accumulation compared with the recruitment of circulating monocytes in established atherosclerotic lesions [46]. Therefore, *Swp*^−^^*/−*^*ApoE*^*−/−*^ mice need to be generated by *Swp*^*−/−*^ crossbred with *ApoE*^*−/−*^ mice and studied in the future.

The development and progression of atherosclerosis are accompanied by a chronic inflammatory response. Our results showed that swiprosin-1 deficiency in macrophages could inhibit the inflammatory cytokine IL-1β and TNF-α secretion in *ApoE*^−/−^ mice fed with HCD in vivo or in macrophages induced by ox-LDL in vitro. Moreover, swiprosin-1 upregulated in both in vivo model aortic tissues of atherosclerosis and in vitro RAW264.7 cells or cultured mouse peritoneal macrophages induced by ox-LDL. These results were in line with the previous study showing that ectopic expression of swiprosin-1 in mast cells augments PMA/A23187-induced NF-κB promoter activity and cytokine expression including IL-3 and IL-8 [8, 9]. Besides, swiprosin-1 was found to be upregulated in in vivo model tissues of passive cutaneous anaphylaxis and atopic dermatitis [8, 9]. Similarly, our previous study also showed swiprosin-1 depletion attenuated inflammatory cytokine production (including IL-1β, IL-6, TNF-α, IL-10, and IFN-γ) in the bronchoalveolar lavage fluid, the kidney homogenates, or the supernatant of peritoneal macrophages isolated from the swiprosin-1 KO mice after LPS treatment [19]. Taken together, these findings indicated that swiprosin-1 upregulated in a number of acute or chronic inflammatory diseases such as atherosclerosis and was involved in cytokine production. Considering that inflammation is an important contributor to the progression and destabilization of the atherosclerotic plaque; thus, the role of swiprosin-1 in atherosclerotic plaque stabilization deserves further study.

The migration ability of macrophages is an important factor affecting atherosclerosis. Swiprosin-1 was identified as an actin-bundling protein-regulating cell spreading and migration in lymphocytes, human mast cells, NK-like cells, and melanoma cells [8, 14]. Moreover, our previous study has reported that deletion of swiprosin-1 neutralized macrophage migration induced by LPS and purified swiprosin-1 could directly promote actin polymerization in vitro [28].

Unexpectedly, our study also found that swiprosin-1 KO in macrophages declined the migration ability in vitro. It was suggested that swiprosin-1 may have a regulatory effect on the accumulation of macrophages in plaques. In the early stage of atherosclerosis, the increase of migration ability of macrophages and the increase of the number of macrophage migration into the subintima of the artery will accelerate the progress of atherosclerosis. In the later stage, the migration of macrophages from the plaque contributes to the regression of the plaque, improves plaque stability, and promotes the improvement of atherosclerosis [47]. Our results showed that swiprosin-1 deficiency decreased the content of macrophages in plaque in ApoE^−/−^ mice fed with HCD for 16 weeks. However, in the above cases, it was unclear that the decrease of the content of macrophages in the plaque was due to the decrease of the number of cells that migrate into plaque in the early stage of atherosclerosis or the increase of the number of cells that migrate out of plaque in the later stage of atherosclerosis. Therefore, the effect of swiprosin-1 on promoting macrophage migration in the development of atherosclerosis needs further study.

Autophagy is a self-protecting cellular catabolic pathway. Macrophage autophagy has been shown to be protective against atherosclerosis [4]. Accumulating evidences demonstrated that macrophage autophagy plays an atheroprotective role in inhibiting inflammation and apoptosis and in promoting cholesterol efflux [48]. Our results showed that the expression of autophagy-related protein LC3B increased and P62 decreased in swiprosin-1 KO macrophages compared with the control macrophages induced by ox-LDL, indicating that swiprosin-1 deficiency could promote autophagy in macrophages. Therefore, the results were consistent with previous results that swiprosin-1 deficiency in macrophages inhibited inflammation and apoptosis and promoted cholesterol efflux. However, the more detailed molecular mechanisms of how swiprosin-1 regulates macrophage autophagy warrant further study.

In short, our study demonstrated that swiprosin-1 deficiency in macrophages could attenuate the development and progression of atherosclerosis. The proatherogenic effects of swiprosin-1 in macrophages might involve inhibition of autophagy and the cholesterol efflux, and acceleration of apoptosis, migration, and the secretion of inflammatory factors. Therefore, swiprosin-1 antagonists may hold significant translational potential for ameliorating atherosclerosis in the future.

## Supplementary information

SUPPLEMENTAL MATERIAL

Supplement Figure 1

Supplement Figure 2

## Data Availability

The data generated or analyzed during this study are included in this article, or if absent are available from the corresponding author upon reasonable request.
