# Supplementary material for: Swiprosin-1 deficiency in macrophages alleviated atherogenesis
Source: Cell Death Discov. 2021 Nov 10;7:344. doi: 10.1038/s41420-021-00739-y (PMC8580969; doi:10.1038/s41420-021-00739-y)
Supplement: Supplementary file 1 — SUPPLEMENTAL MATERIAL [file 41420_2021_739_MOESM1_ESM.docx]

**Swiprosin-1 deficiency in macrophages alleviated atherogenesis**

Ling-Chang Tong^a,b,1^, Zhi-Bin Wang^b,1^, Jia-Qi Zhang^a,1^, Yue Wang^b,c^, Wei-Ye Liu^b,^, Hao Yin^a^, Jia-Cheng Li^a^, Ding-Feng Su^b^, Yong-Bing Cao^a,*^, Li-Chao Zhang^c,*^, ing Li^a,b,*^

^a^ *Shanghai TCM-Integrated Institute of Vascular Disease, Shanghai TCM- Integrated Hospital, Shanghai University of Traditional Chinese Medicine, Shanghai, China*

^b^ *Department of Pharmacology, College of Pharmacy, Second Military Medical University, Shanghai, China*

^c^ *Department of Pharmacy, Shanghai Municipal Hospital of Traditional Chinese Medicine, Shanghai, China*

^1^These authors contributed equally to this work.

Short title: Swiprosin-1 deficiency in macrophages alleviated atherosclerosis

^#^*Corresponding authors at*: Institute of Vascular Disease, Shanghai TCM- Integrated Hospital, Shanghai University of Traditional Chinese Medicine, Shanghai 200082, China.

E-mail: ybcao@vip.sina.com (Y.-B. Cao) and changhaiskin@163.com (L.-C. Zhang)

and lingli_z163@163.com (L. Li)

**Supplement 1**

**
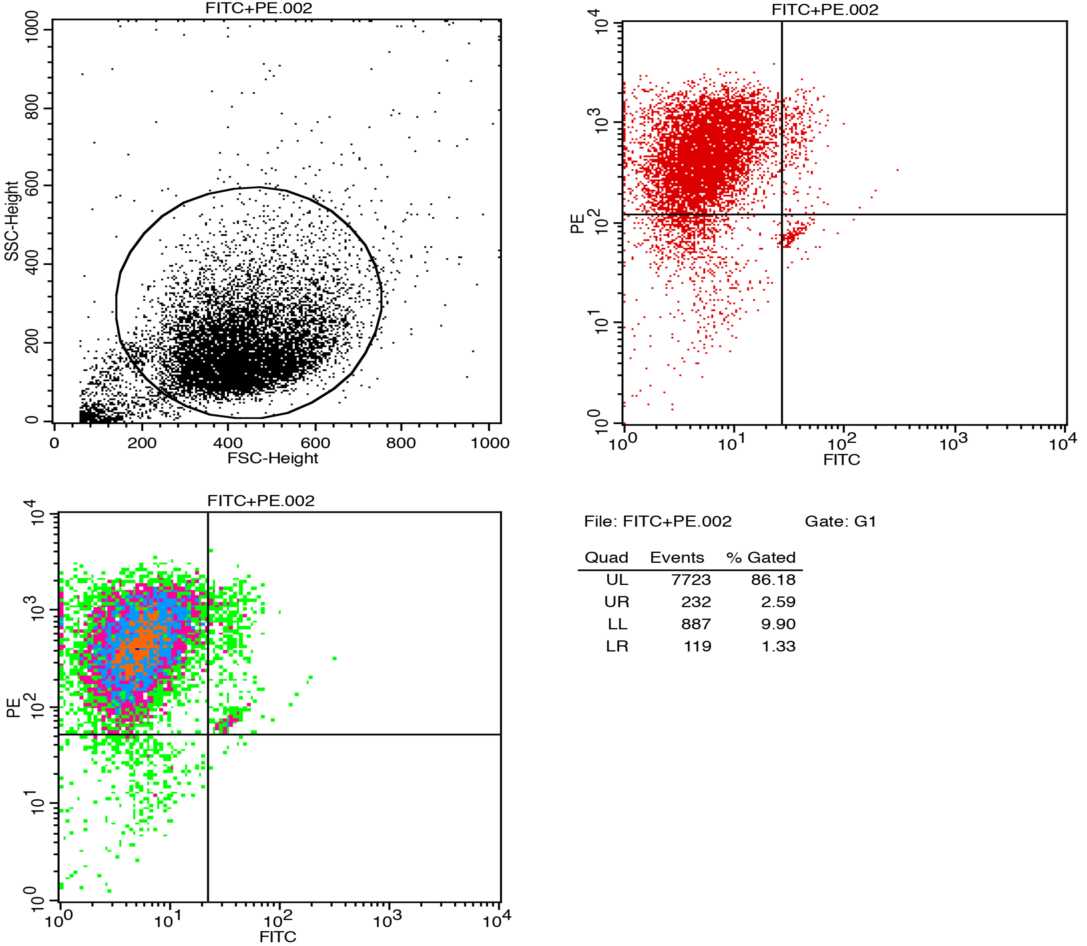
**

Flow cytometry of results showed the primary peritoneal macrophages labeled with anti-F4/80 antibody were successfully isolated from mice.

**Supplement 2**


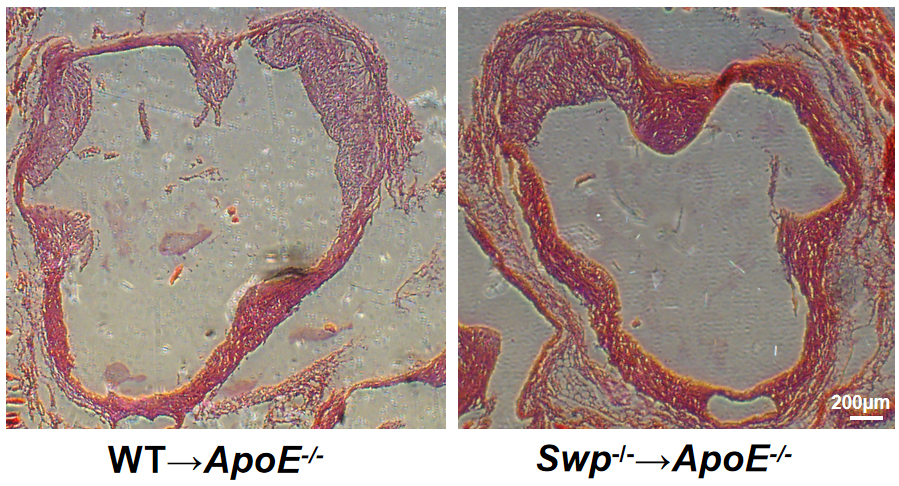


Representative images of aortic root sections from *Swp^-/-^→ApoE^-/-^* and WT→*ApoE^-/-^* mice stained with Hematoxylin and eosin staining. Scale bars are 200 μm.
