# Supplementary figures and images for: Swiprosin-1 deficiency in macrophages alleviated atherogenesis
Source: Cell Death Discov. 2021 Nov 10;7:344. doi: 10.1038/s41420-021-00739-y (PMC8580969; doi:10.1038/s41420-021-00739-y)

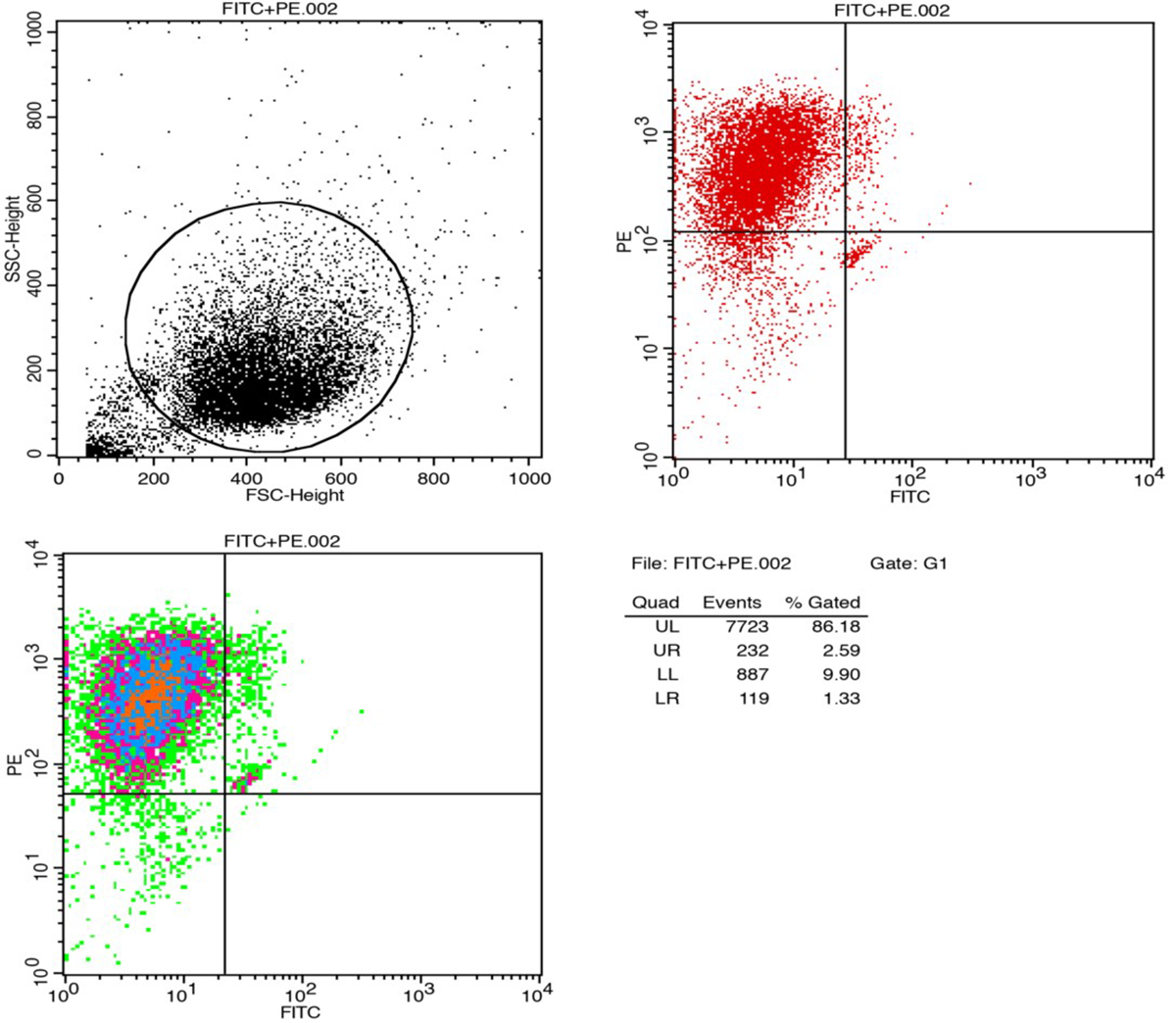

Supplement: Supplementary file 2 — Supplement Figure 1 [file 41420_2021_739_MOESM2_ESM.tif]

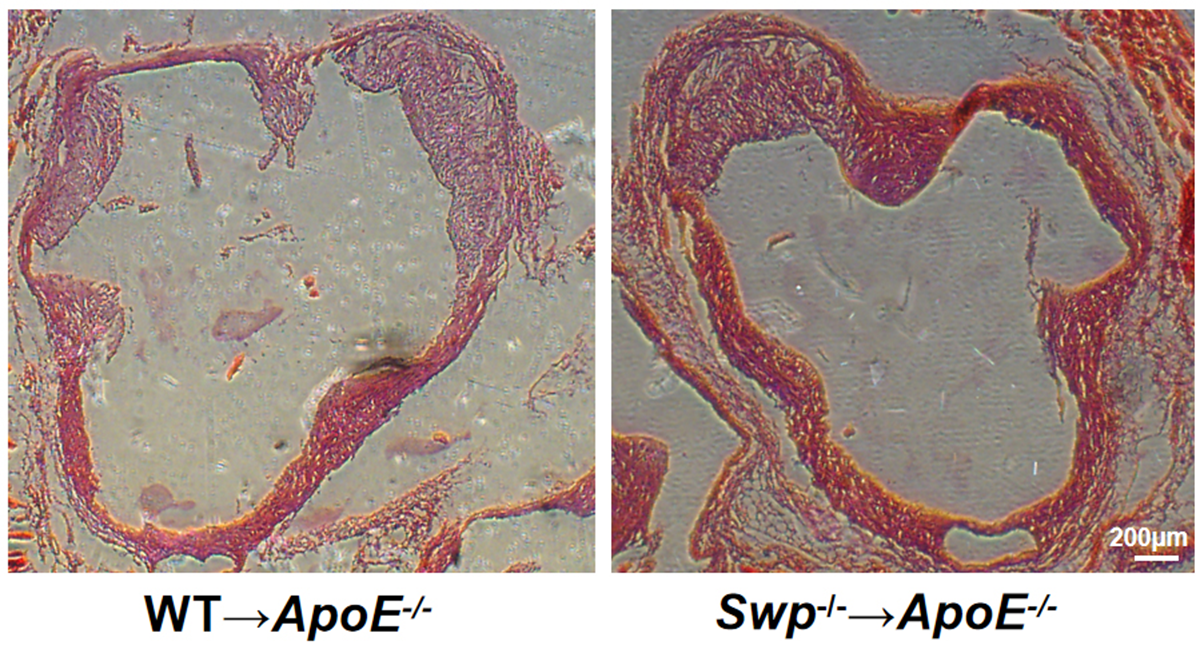

Supplement: Supplementary file 3 — Supplement Figure 2 [file 41420_2021_739_MOESM3_ESM.tif]
